# Evidence of Impact: iCCM as a strategy to save lives of children under five

**DOI:** 10.7189/jogh.09.010801

**Published:** 2019-06

**Authors:** Debra Prosnitz, Samantha Herrera, Helen Coelho, Lwendo Moonzwe Davis, Kirsten Zalisk, Jennifer Yourkavitch

**Affiliations:** 1 ICF, Rockville, Maryland, USA; 2 Save the Children, Washington, D.C., USA

## Abstract

**Background:**

In 2013, the World Health Organization (WHO) launched the Rapid Access Expansion (RAcE) programme in the Democratic Republic of Congo, Malawi, Mozambique, Niger, and Nigeria to increase coverage of diagnostic, treatment, and referral services for malaria, pneumonia, and diarrhea among children ages 2-59 months. In 2017, a final evaluation of the six RAcE sites was conducted to determine whether the programme goal was reached. A key evaluation objective was to estimate the reduction in childhood mortality and the number of under-five lives saved over the project period in the RAcE project areas.

**Methods:**

The Lives Saved Tool (LiST) was used to estimate reductions in all-cause child mortality due to changes in coverage of treatment for the integrated community case management (iCCM) illnesses – malaria, pneumonia, and diarrhea – while accounting for other changes in maternal and child health interventions in each RAcE project area. Data from RAcE baseline and endline household surveys, Demographic and Health Surveys, and routine health service data were used in each LiST model. The models yielded estimated change in under-five mortality rates, and estimated number of lives saved per year by malaria, pneumonia and diarrhea treatment. We adjusted the results to estimate the number of lives saved by community health worker (CHW)-provided treatment.

**Results:**

The LiST model accounts for coverage changes in iCCM intervention coverage and other health trends in each project area to estimate mortality reduction and child lives saved. Under five mortality declined in all six RAcE sites, with an average decline of 10 percent. An estimated 6200 under-five lives were saved by malaria, pneumonia, and diarrhea treatment in the DRC, Malawi, Niger, and Nigeria, of which approximately 4940 (75 percent) were saved by treatment provided by CHWs. This total excludes Mozambique, where there were no estimated under-five lives saved likely due to widespread stockouts of key medications. In all other project areas, lives saved by CHW-provided treatment contributed substantially to the estimated decline in under-five mortality.

**Conclusions:**

Our results suggest that iCCM is a strategy that can save lives and measurably decrease child mortality in settings where access to health facility services is low and adequate resources for iCCM implementation are provided for CHW services.

Integrated community case management (iCCM) is an equity-focused strategy to provide timely treatment for common childhood illness at the community level by community health workers (CHWs) [1]. The iCCM strategy targets hard-to reach-areas where access to care is typically a challenge, defined in most contexts as communities more than five kilometers from a functional health facility. iCCM targets three common, treatable, and curable childhood illnesses that account for high rates of mortality and morbidity and exert a significant burden on the health system: malaria, pneumonia, and diarrhea. iCCM interventions include diagnostic, treatment, and referral services for malaria, pneumonia, and diarrhea. The implementation of iCCM interventions has been demonstrated to increase prompt and appropriate treatment in children under five years of age [2-5].

In 2013, the World Health Organization (WHO) launched the Rapid Access Expansion (RAcE) programme in the Democratic Republic of the Congo (DRC), Malawi, Mozambique, Niger, and Nigeria to increase coverage of iCCM interventions among children ages 2-59 months. Two projects were implemented in Nigeria; one in Abia State and one in Niger State. Through training, deploying, equipping, and supervising CHWs, RAcE aimed to achieve universal iCCM coverage in designated hard-to-reach areas supported by the programme. iCCM-trained CHWs in RAcE project areas assessed, diagnosed, and treated or referred as appropriate, children with malaria, pneumonia, and diarrhea. Malaria was diagnosed using rapid diagnostic tests (RDTs) and treated with artemisinin combination therapy (ACT); pneumonia was diagnosed by observation of difficult breathing and by counting respiratory rate for age and treated with amoxicillin; diarrhea was treated with oral rehydration solution (ORS) and zinc. iCCM protocols aligned with the national policy in each country supported this case management by CHWs. While RDTs were used to diagnose malaria in most project areas for the duration of RAcE implementation, in Malawi RDTs were rolled out mid-project, resulting in a change in malaria treatment protocol from presumptive treatment to confirmed treatment.

RAcE projects were implemented by international non-governmental organizations (NGOs) in partnership with Ministries of Health (MOH) and WHO. Each project’s area of implementation, target population, implementing partners, and period of performance are shown in **Table 1**. In RAcE project areas in the DRC, Niger, and Nigeria (Abia and Niger States), iCCM was introduced through the RAcE projects and operated in an environment with few other child health interventions.

**Table 1:** RAcE project areas of implementation, target populations, implementing partners, and period of performance

RAcE Project	Area of implementation	Target population	Implementing partners	Period of performance
DRC	7 health zones in Tanganyika Province in 2013; expanded to 11 health zones by 2016	Estimated total population of 1 000 000 including 150 000 children under 5 years of age	International Rescue Committee, DRC Ministry of Public Health (MOPH), WHO	September 2013 – November 2017
Malawi	Dedza, Mzimba North, Ntcheu, and Ntchisi districts in 2013; expanded to Likoma, Lilongwe Rural, Nhkata Bay, and Rumphi districts in 2014	Estimated total population of 1 625 036 including 276 256 children under five years of age	Save the Children, Malawi Ministry of Health (MOH), D-Tree International, Medical Care Development International, WHO	April 2013 – September 2017
Mozambique	Inhambane, Manica, Nampula, and Zambezia provinces	Estimated total population of 4 196 074 including 719 444 children under 5 years of age	Save the Children, Malaria Consortium, Mozambique Ministry of Health (*MISAU*), WHO, UNICEF, World Bank	April 2013 – December 2016
Niger	3 health districts in Dosso Region (Boboye, Dogondoutchi, and Dosso) and 1 health district in Tahoua region (Keita)	Estimated total population of 994 904, including 230 833 children under five years of age	World Vision, Niger Ministry of Public Health (MSP), WHO	July 2013 – September 2017
Nigeria – Abia State	15 local government areas (LGAs): Arochukwu, Bende, Ikwuano, Isialangwa North, Isialangwa South, Isuikwuato, Nneochi, Obingwa, Ohafia, Osisioma, Ugwunagbo, Ukwa East, Ukwa West, Umuahia North, and Umuahia South	Estimated total population of 1 268 738, including 202 998 are children under 5 years of age	Society for Family Health (SFH), Abia State Primary Health Care Development Agency (PHCDA), Abia State MOH, Grassroots Community Development Initiative, Population Services International, and the Institute of Tropical Diseases Research and Prevention at the University of Calabar, Nigeria, WHO	November 2013 – December 2017
Nigeria – Niger State	Six LGAs: Edati, Lapai, Mariga, Paikoro, Rafi, and Rijau	Estimated total population of 814 845 including 161 973 children under 5 years of age	Malaria Consortium, Niger State MOH, Niger State PHCDA	November 2013 – December 2017

RAcE – Rapid Access Expansion, LGA – local government area, DRC – the Democratic Republic of the Congo

In 2017, ICF conducted final evaluations of each of the six RAcE project areas to determine whether the programme goal of increased coverage of diagnostic, treatment, and referral services for malaria, pneumonia, and diarrhea to decrease overall child mortality was reached. One of the main evaluation objectives was to estimate the reduction in childhood mortality, and number of under-five lives saved over the project period in the RAcE project areas.

## METHODS

We used the Lives Saved Tool (LiST) to estimate the impact of changes in coverage of treatment for the three illnesses targeted by iCCM – malaria, pneumonia, and diarrhea – while accounting for other changes in maternal and child health interventions in each RAcE project area.

LiST is computer-based software for modeling maternal and child mortality that runs through Spectrum. Spectrum is a suite of eleven integrated modules built and maintained by Avenir Health under guidance of a team at Johns Hopkins Bloomberg School of Public Health’s Institute for International Programs [6-8]. Included in the Spectrum suite of modules are DemProj (demography), AIM (AIDS impact model), FamPlan (family planning), and LiST (child survival). LiST estimates the impact of scaling up of maternal, newborn, and child health and nutrition intervention coverage. Specifically, LiST estimates all-cause child mortality and the number of child lives saved by intervention and by cause. To produce these estimates, LiST incorporates detailed demographic information through automated linkages with the DemProj and FamPlan modules; country-specific cause of death information for newborns, children under five, and mothers; health and nutrition status, including through linkage with the AIM module; coverage of key health interventions; and effectiveness measures for each intervention determined by scientific review [9-11]. LiST Version 5.55 was used for this study.

We created six LiST models, one for each RAcE project area. Each LiST model was built from the country’s DemProj data and national mortality profiles in Spectrum. The under-five cause of death information for each country, which includes the three iCCM focus illnesses, is shown in **Table 2**.

**Table 2:** Cause-specific mortality for under-five (ages 1-59 months) mortality by country, from Spectrum

Country	Diarrhea	Pneumonia	Malaria	Meningitis	Measles	Pertussis	AIDS	Injury	Other
DRC	14.1%	22.8%	17.9%	4.1%	7.1%	1.6%	0.7%	6.6%	25.2%
Malawi	13.1%	18.8%	19.5%	3.7%	4.1%	1.9%	7.9%	6.7%	24.4%
Mozambique	11.6%	25.3%	13.4%	7.4%	0.3%	2.3%	4.5%	8.3%	27.0%
Niger	14.6%	31.5%	10.9%	7.8%	0.04%	1.2%	0.2%	8.8%	25.1%
Nigeria	14.3%	29.1%	18.3%	3.8%	2.5%	0.5%	1.0%	7.0%	23.6%

DRC – the Democratic Republic of the Congo

Using LiST’s subnational projection module, we adjusted each model to reflect the RAcE project area population, baseline subnational mortality rates (as available), and baseline intervention coverage estimates. This adjustment relies on a comparison between national (default data in Spectrum modules) and subnational data [12]. To accurately make this comparative adjustment, national data must be comparable to subnational data. If data were from the same source, then the data were comparable, and the subnational data point was input into the model. If subnational data were from a different data source than the source of national data in LiST, we adjusted these by creating a ratio between the subnational coverage rate and the national coverage rate, and then applying this ratio to the national coverage rate given in the subnational LiST module [12].

We used data from RAcE baseline and endline household surveys, Demographic and Health Surveys (DHS), WHO-UNICEF, and routine health service data as inputs for each LiST model.

Baseline years were set to 2013 for the DRC, Mozambique, Niger, and the two Nigeria models. For Malawi, the baseline year was set to 2010 in concurrence with the available baseline DHS. The subnational baseline files were used to create the full models in LiST.

Data for treatment of pneumonia with amoxicillin, treatment of fever with ACT within 48 hours, treatment of diarrhea with ORS, and treatment of diarrhea with zinc from the RAcE baseline and endline household surveys were input into each LiST model for baseline and endline data points, respectively. For Malawi, 2010 DHS data for treatment of pneumonia with amoxicillin, fever with ACT, and diarrhea with ORS and zinc were held constant to 2013 values, and 2013 RAcE baseline household survey data points were input for 2013. iCCM intervention coverage modeled is summarized in **Table 3**.

**Table 3:** iCCM intervention coverage in RAcE Project areas

iCCM intervention coverage	DRC		Malawi		Mozambique		Niger		Nigeria – Abia State		Nigeria – Niger State	
**Baseline**	**Endline**	**Baseline**	**Endline**	**Baseline**	**Endline**	**Baseline**	**Endline**	**Baseline**	**Endline**	**Baseline**	**Endline**
ORS for diarrhea	48.9 (40.9-57.0)	71.7 (63.1-79.0)	72.3 (64.9-78.7)	72.6 (65.3-78.9)	69.8 (60.9-77.5)	69.9 (62.5-76.5)	61.7 (53.8-68.9)	78.4 (69.3-85.4)	31.4 (25.7-37.8)	55.4 (46.6-63.9)	68.2 (59.5-75.7)	88.3 (81.9-92.7)
Zinc for diarrhea	2.9 (1.5-5.5)	58.8 (48.6-68.3)	23.8 (17.4-31.5)	28.3 (21.2-36.6)	9.9 (6.1-15.6)	35.0 (25.2-46.4)	30.4 (24.3-37.2)	72.6 (65.7-78.5)	7.2 (4.2-12.2)	42.0 (34.7-49.6)	15.0 (10.5-20.9)	77.0 (66.8-84.8)
Careseeking for pneumonia	17.9 (13.1-23.8)	53.0 (42.8-63.0)	56.5 (48.2-64.3)	66.8 (59.0-73.8)	69.6 (61.1-76.9)	58.8 (50.9-66.2)	44.6 (36.5-52.9)	46.2 (36.3-56.5)	8.6 (5.3-13.6)	35.5 (28.3-43.5)	28.6 (21.6-36.7)	60.5 (50.2-69.9)
ACTs for fever within 48 h	5.2 (3.0-8.7)	49.6 (41.5-57.8)	43.5 (36.4-50.9)	-*	55.0 (48.4-61.4)	39.7 (33.5-46.3)	41.9 (34.1-50.1)	58.4 (47.4-68.5)	15.3 (11.9-19.4)	38.3 (33.5-43.3)	27.4 (22.1-33.3)	64.5 (56.3-71.9)

iCCM – integrated community case management, DRC – the Democratic Republic of the Congo, ACT – artemisinin combination therapy, ORS – oral rehydration solution

*Due to the change in Malawi’s iCCM treatment policy, from presumptive treatment to confirmed treatment of malaria, the percentage of cases of fever treated with ACT within 48 hours measured at baseline was held constant in the model to avoid an artificial decrease in treatment coverage resulting in lives lost.

Baseline and endline data points for other maternal, newborn, and child health (MNCH) interventions input into the model were sub-national DHS data, where available. The lowest administrative level (eg, state, province, or district) of DHS data available comprising the project area was used. WHO and UNICEF estimates of immunization coverage were used in all countries except Malawi, for which DHS estimates were used. No large-scale national-level survey data for 2016 were available for the DRC, Mozambique, Niger, and Nigeria at the time of the RAcE final evaluation. Routine health service data for Mozambique project areas in 2016 were used for that model’s endline; if data were unavailable, values were held constant. For the DRC and Nigeria, we projected 2016 estimates for MNCH indicators by applying linear interpolation to continue the trend as measured by DHS for the time period preceding the 2013 baseline. For Niger, available routine health service data were used for 2016 and linear interpolation of DHS data was used to estimate other indicators values. Thus, the models assumed that the same trends measured for the preceding time period continued through 2016.

Values for all indicators were linearly interpolated from 2013 to 2016 in each model, except where otherwise noted. If projected values were over 100 percent, these were determined unrealistic and consequently baseline values were held constant. Due to the change in Malawi’s iCCM treatment policy, from presumptive treatment to confirmed treatment of malaria, the percentage of cases of fever treated with ACT within 48 hours measured at baseline was held constant in the model to avoid an artificial decrease in treatment coverage resulting in lives lost. A summary of data sources for the LiST models is provided in **Table 4**.The data, and their sources, used to create each project’s LiST model are detailed in Tables S1-S6 in the **Online Supplementary Document****.**

**Table 4:** Summary of data sources for Lives Saved Tool (LiST) models

	LiST data sources		
**LiST default**	**Baseline**	**Endline**
**DemProj**			
Population	United Nations Population Division	Estimated by RAcE grantee adjusted with ratio	Calculated by model
Total fertility rate	DHS	DHS adjusted with ratio	Calculated by model
**AIM**			
HIV incidence or prevalence	DHS	LiST default or DHS adjusted with ratio	Calculated by model
**FamPlan**			
Contraceptive prevalence rate	DHS	DHS adjusted with ratio	Calculated by model
**LiST**			
**Baseline child mortality**			
Neonatal mortality rate	DHS	DHS	Calculated by model
Infant mortality rate	DHS	DHS	Calculated by model
Under-five mortality rate	DHS	DHS	Calculated by model
**Pregnancy**			
Antenatal care	DHS	DHS or routine health service data	DHS* or routine health service data
Tetanus toxoid vaccine	WHO-UNICEF	DHS or routine health service data adjusted with ratio	DHS* or routine health service data
Intermittent preventive treatment of malaria during pregnancy	DHS	DHS or routine health service data	DHS* or routine health service data
Multiple micronutrition supplementation (iron folate 90+)	DHS	DHS or routine health service data	DHS* or routine health service data
**Childbirth**			
Skilled birth attendance	DHS	DHS†	DHS* or routine health service data †
Institutional delivery	DHS	DHS or routine health service data	DHS* or routine health service data
**Preventive**			
Chlorohexadine for postnatal	N/A	LiST default, routine health service data, or N/A	DHS, routine health service data, or N/A
Postnatal care (“clean postnatal practices”)	DHS	LiST default	N/A
Vitamin A supplementation	DHS	DHS	DHS*
Improved water source	WHO-UNICEF	DHS	DHS* or N/A
Water connection in home	WHO-UNICEF	LiST default or DHS	DHS* or N/A
Improved sanitation	WHO-UNICEF	DHS	DHS* or N/A
Hygienic disposal of child's stools	DHS	DHS or N/A	DHS* or N/A
Insecticide-treated net ownership	DHS	DHS	DHS*, MIS, or N/A
**Vaccines**			
Bacille Calmette-Guerin (BCG) vaccine	WHO-UNICEF	WHO-UNICEF or DHS	WHO-UNICEF or DHS
Diphtheria, pertussis, and tetanus (DPT) vaccine	WHO-UNICEF	WHO-UNICEF or DHS	WHO-UNICEF or DHS
Haemophilus influenza type b (Hib) vaccine	WHO-UNICEF	WHO-UNICEF or DHS	WHO-UNICEF or DHS
Hepatitis B vaccine	WHO-UNICEF	DHS or N/A	DHS or N/A
Measles vaccine	WHO-UNICEF	WHO-UNICEF or DHS	WHO-UNICEF or DHS
Polio vaccine	WHO-UNICEF	WHO-UNICEF or DHS	WHO-UNICEF or DHS
Pneumococcal vaccine	WHO-UNICEF	WHO-UNICEF or DHS	WHO-UNICEF or DHS
Rotavirus vaccine	WHO-UNICEF	WHO-UNICEF or DHS	WHO-UNICEF or DHS
**Curative**			
ORS for diarrhea	DHS	RAcE baseline survey	RAcE endline survey
Antibiotics for diarrhea	DHS	DHS, routine health service data, or N/A	DHS*, routine health service data, or N/A
Zinc for diarrhea	DHS	RAcE baseline survey	RAcE endline survey
Antibiotics for cough with fast or difficult breathing	DHS	RAcE baseline survey	RAcE endline survey
ACT for fever same or next day	DHS	RAcE baseline survey	RAcE endline survey

ACT – artemisinin combination therapy; DHS – Demographic Health Survey; MIS – Malaria Indicator Survey; ORS – oral rehydration solution, N/A – not available; when endline data was not available, the baseline value was held constant over time

*DHS data point input or obtained by extrapolating pre-baseline DHS trend.

†Set to same value as institutional delivery.

Each LiST model yielded three outputs: 1) Under-five mortality rates for each year, to estimate the change in all-cause child mortality from baseline to endline (estimated impact); 2) Number of lives saved per year, among children under five years of age; and 3) Number of lives saved per year by intervention, including malaria treatment, pneumonia treatment, diarrhea treatment with zinc, and diarrhea treatment with ORS. The number of lives saved by malaria treatment, pneumonia treatment, and diarrhea treatment in each RAcE project area model were then adjusted proportionally to the percentage of cases treated by CHWs as measured in the respective RAcE endline household surveys to provide an estimate of the lives saved due to iCCM services in the project areas. Because the LiST model uses the coverage estimates for the baseline year to estimate lives saved for the model’s projection period, the estimated lives saved for the baseline year is, by default, zero.

## RESULTS

Under-five mortality declined in all six RAcE sites over the course of the programme, with an average decline of 10 percent. An estimated 6200 under-five lives were saved by malaria, pneumonia, and diarrhea treatment in the DRC, Malawi, Niger, and Nigeria. Approximately 4940 (75 percent) of under-five lives were saved by treatment provided by CHWs. This total excludes Mozambique, where there were no estimated under-five lives saved. The total estimated lives saved – lives saved by pneumonia, diarrhea, and malaria treatment in – each project area are shown in **Table 5**. The proportion of estimated lives saved by iCCM treatment from CHWs in each RAcE project area are shown in **Table 6**. Estimates of lives saved in each project area by year and by treatment intervention are provided in Tables S7-S12 in the **Online Supplementary Document**.

**Table 5:** Estimated lives saved by pneumonia, diarrhea, and malaria treatment by all providers, estimated under-five mortality decrease in RAcE project areas, and estimated under-five mortality decrease

RAcE Project	Estimated U5 lives saved (net)	Estimated U5 lives saved by pneumonia treatment	Estimated U5 lives saved by diarrhea treatment (ORS and zinc)	Estimated U5 lives saved by malaria treatment	Estimated % decrease in U5MR
DRC	1855	493	579	743	15.2
Malawi	3,161	508	64	0^†^	4.6
Mozambique	-95	-912	268	-1675	0.2
Niger	1931	54	747	327	12.6
Nigeria – Abia State	1,573	472	455	480	12.1
Nigeria – Niger State	1298	407	361	506	14.5
Total	**9723**	**1022**	**2474**	**381**	**Average: 9.9**

N/A – not applicable, U5 – under-five, iCCM – integrated community case management, ORS – oral rehydration solution, U5MR – under-five mortality rate, DRC – the Democratic Republic of the Congo, RAcE – Rapid Access Expansion

†Malaria treatment coverage was not modeled for Malawi.

**Table 6:** Estimated lives saved by CHW-provided treatment in RAcE project areas

RAcE Project	Estimated U5 Lives Saved by CHW-provided pneumonia, diarrhea, and malaria treatment (iCCM)
DRC	1728
Malawi	216
Mozambique	N/A
Niger	965
Nigeria – Abia State	967
Nigeria – Niger State	1062
Total	**4938**

N/A – not applicable, U5 – under-five, CHW – community health worker, iCCM – integrated community case management, DRC – the Democratic Republic of the Congo, RAcE – Rapid Access Expansion

In the DRC, there was an estimated 15.2 percent decrease in under-five mortality over the course of the project. An estimated total of 1820 deaths were prevented (lives saved) by pneumonia, diarrhea, and malaria treatment interventions in children under 5 years of age over the course of the project. Based on the coverage of treatment by CHWs, an estimated 1730 (95 percent) of these lives were saved due to treatment provided by CHWs.

In Malawi, there was an estimated 4.6 percent decrease in under-five mortality over the course of the project. An estimated 570 deaths were prevented by pneumonia and diarrhea treatment interventions in children under 5 years of age over the course of the project. There were no estimated deaths prevented by malaria treatment. Based on the coverage of treatment by CHWs, an estimated 220 (38 percent) of these lives were saved due to treatment provided by CHWs.

In Mozambique, there was an estimated 0.2 percent decrease in under-five mortality over the course of the project. An estimated 100 child lives were lost in RAcE project areas over the project period. This estimate accounts for significant numbers of lives saved by increases in some interventions and lives lost due to decreases in coverage of others. An estimated 250 lives were saved due to the increase in diarrhea treatment coverage of zinc, and 20 lives due to the increase in ORS treatment coverage. An estimated 910 lives were lost due to a decrease in treatment coverage of pneumonia with oral antibiotics, and 1680 due to a decrease in treatment coverage of malaria with ACTs.

In Niger, there was an estimated 12.6 percent decrease in under-five mortality over the course of the project. An estimated 1130 deaths were prevented by pneumonia, diarrhea, and malaria treatment interventions in children under five years of age over the course of the project. Based on the coverage of treatment by CHWs, an estimated 970 (86 percent) of these lives were saved by treatments provided by CHWs.

In Abia State, Nigeria there was an estimated 12.1 percent decrease in under-five mortality over the course of the project. An estimated 1400 deaths were prevented by pneumonia, diarrhea, and malaria treatment interventions in children under five years of age over the course of the project. Based on the coverage of treatment by CHWs, an estimated 970 (69 percent) of these lives were saved by treatments provided by CHWs.

In Niger State, Nigeria there was an estimated 14.5 percent decrease in under-five mortality over the course of the project. An estimated 1280 deaths were prevented by pneumonia, diarrhea, and malaria treatment interventions in children under five years of age over the course of the project. Based on the coverage of treatment by CHWs, an estimated 1100 (83 percent) of these lives were saved by treatments provided by CHWs.

## DISCUSSION

Our results reflect the observed coverage changes measured in each project area and, with LiST, show estimated mortality reductions in a way that can be adjusted to explain the impact of each iCCM project amidst other ongoing health trends [13]. We found an average estimated decline in under-five mortality of 9.9 percent across the six project areas, ranging from a 0.2 percent decline in Mozambique to a 15.2 percent decline in the DRC. An estimated 1020 child lives were saved from pneumonia treatment; an estimated 2470 child lives saved from diarrhea treatment; and an estimated 380 child lives saved from malaria treatment across the six project areas. Our findings show that when adjusted to reflect the proportion of treatment coverage provided by CHWs vis-à-vis treatment coverage by all providers, a substantial number of estimated child lives were saved by CHW-provided treatment. In the DRC, approximately 95 percent of the child lives were saved by CHW-provided treatment. In Niger and Niger State, Nigeria CHW-provided treatment accounted for over 80 percent of the child lives saved, and in Abia State nearly 70 percent. In Malawi, less than 50 percent of child lives were saved by CHW-provided treatment; the percentage in Mozambique is unclear because it could not be accounted for in the model.

Previous studies have used LiST to retrospectively evaluate changes in mortality and deaths averted by high-impact child health interventions [14-17]. LiST has also been used to demonstrate decreases in mortality by NGO-facilitated projects that used community-based intervention packages to contribute to improved coverage of child health interventions [18], as well as to demonstrate the potential impact of expanding coverage of such interventions through scaling up CHW-delivered interventions at the population level [19]. An evaluation of iCCM in Burkina Faso also used LiST to model mortality changes, and, similar to our study, used program data to inform the interpretations of both changes in mortality and changes in coverage measured through household surveys [17]. Two evaluations of the Catalytic Initiative in Malawi demonstrated the impact of iCCM, one using LiST. The first used population-based survey data with results showing a significant decrease in child mortality over the period of implementation, and that the majority of deaths averted were due to interventions that could be delivered at the community level by CHWs [14]. In contrast to our analysis, this study did not examine the proportion of these interventions actually delivered by CHWs. The second drew conclusions about a decrease in mortality using national level changes in mortality estimates from DHS and Multiple Indicator Cluster Survey (MICS), but did not find evidence that these increases in intervention coverage were due to iCCM [15]. Similar to our findings, contextual information suggested that, for a number of reasons, only a small proportion of treatment of malaria, pneumonia, and diarrhea is provided by CHWs in Malawi [15]. A similar evaluation of iCCM in Uganda that utilized household survey data in LiST found that iCCM increased treatment coverage for diarrhea and fever, mitigated effects of amoxicillin stockouts for pneumonia treatment, and saved lives [16]. While that study supports the potential impact of iCCM, it did not account for changes in other MNCH interventions during the study period, as our analysis did.

Substantial increases in the iCCM intervention coverage were measured from baseline to endline in the DRC, Niger, and Nigeria project areas, with high proportions of cases treated by CHWs. Small, non-significant increases were measured in diarrhea and pneumonia treatment in Malawi project areas. Holding the percentage of cases of fever treated with ACT within 48 hours measured at baseline constant in the Malawi model, to account for the change in iCCM treatment policy and to avoid an artificial decrease in treatment coverage resulting in lives lost, resulted in the model showing no estimated impact from malaria treatment. The Malawi model likely underestimated the number of child lives saved due to iCCM intervention coverage, because it does not account for lives saved by the improvement in malaria case management following the introduction of RDTs.

With the exception of Mozambique, overall coverage of pneumonia, malaria, and diarrhea treatment increased in each RAcE project area, including measurable increases in treatments provided both at the facility level and by CHWs in communities. Unlike other RAcE project sites, there were measured decreases in overall malaria and pneumonia treatment coverage – treatment by any provider – in Mozambique. Diarrhea treatment with ORS did not significantly change, while diarrhea treatment with zinc increased significantly. Due to the decreases in pneumonia and malaria treatment coverage, the LiST model estimated that lives were lost. However, the source of treatment among pneumonia cases that received treatment shifted over time – with a larger proportion being treated by CHWs at endline; the model obscured this effect of pneumonia treatment provided by CHWs. There was not a statistically significant increase in the proportion of malaria cases treated by CHWs, likely because of widespread stockouts of malaria kits. Thus, there were likely not a significant number of lives saved due to CHW-provided malaria treatment in Mozambique. The largest decreases in mortality and numbers of lives saved (estimated impact) were in project areas in which iCCM was introduced through RAcE (the DRC, Niger, and Nigeria). Given that, in these contexts, RAcE operated in an environment with few other child health interventions that could have influenced the estimated decreases in under-five mortality, it is likely that iCCM contributed substantially to the estimated mortality decreases. In areas in which RAcE was supporting the continuation and expansion of more a mature iCCM strategy (Malawi and Mozambique), there was less estimated impact. In Mozambique, widespread stockouts due to weaknesses in the health system limited the provision of life saving treatment and consequently, we observed negligible impact on mortality reduction.

The LiST model is a valuable tool for estimating the impact of maternal and child health intervention coverage on child mortality, though not without limitations [13,20]. Direct measurement of child mortality is a time and resource intensive effort, and thus typically done every 5 to 10 years at the national level and highest subnational administrative unit by large-scale surveys such as DHS and MICS. This is a common constraint of sub-national public health programmes, and why models such as LiST can be a useful proxy for estimating impact. Child mortality was not directly measured before or after RAcE project implementation in any country, and valid mortality data were not available for the project sites before or after RAcE.

### Limitations

LiST does not account for the mode of delivery or source of care (with the exception of facility birth). Thus, the model estimates the impact of malaria, pneumonia, and diarrhea treatment based on RAcE project data measured in RAcE household surveys. For all other interventions, except immunization, the model and results assume that project areas had projected intervention coverage according to the same trends measured by the DHS or national health information system. The areas represented by these estimates do not align exactly with the RAcE project areas. Additionally, there are potential quality issues with the routine health service data, due to challenges in health management information system reporting. Some models had missing data points, and had values held constant over time or used projected estimates for endline values. WHO and UNICEF national immunization coverage estimates likely overestimated coverage in the hard-to-reach RAcE project areas. Furthermore, the LiST model does not account for changes in diagnostics, the quality of care, timeliness of pneumonia and diarrhea treatment, or referrals made or completed. This study was not designed to directly attribute changes in outcomes or impact to the individual RAcE projects or to the RAcE programme. There are no valid counterfactuals for these analyses. However, RAcE-supported iCCM services delivered by CHWs were the only iCCM services provided in the project areas.

## CONCLUSION

The results of the RAcE LiST models suggest that iCCM is a strategy that can save lives and measurably decrease child mortality in settings where access to health facility services is low and adequate resources, including trained and supported staff and supplies, are provided. The overall increases in child health intervention coverage in RAcE project areas demonstrate the added value of iCCM as an extension of the health system and as an equity-focused strategy.

In the context of this evidence and limited funding for child health, we advocate for continued investment in integrated child health programming. For greatest impact, iCCM should be implemented as an extension of the health system. It is not a strategy to replace health facility services, but rather to extend select, critical life-saving interventions directly to hard-to-reach communities. A successful iCCM programme should be part of a larger effort to strengthen health systems and facility services. The case of Mozambique emphasizes this point in the context of the adage ‘no drugs, no service.’ Without adequate commodities in hand, CHWs are unable to provide life-saving treatment to their communities, and risk losing community trust of their work and the programme.

## Additional Material

Online Supplementary Document
