# Supplementary material for: Evidence of Impact: iCCM as a strategy to save lives of children under five
Source: J Glob Health. 2019 Jun 25;9(1):010801. doi: 10.7189/jogh.09.010801 (PMC6594661; doi:10.7189/jogh.09.010801)
Supplement: Online Supplementary Document [file jogh-09-010801-s001.pdf]

J Glob Health 2019; 9: 010801

Online Supplemental Documents: Evidence of Impact: iCCM as a strategy to save lives of children under five

Summary of LiST Data Inputs for Each RAcE Project Area

Table S1. Intervention coverage indicators and values used in RAcE DRC LiST analysis

RAcE DRC	LiST model baseline inputs—2013			LiST model endline inputs—2016	
	Subnational data	Ratio	Data sources/indicator information	Subnational Data	Data sources/indicator information
DemProj					
Population	1,000,000	1.29%	Project area population estimated by the IRC; adjusted with ratio	-	Calculated by model
Total fertility rate	7.8	1.2	DHS 2013–2014; adjusted with ratio	-	Calculated by model
AIM					
HIV incidence or prevalence	1.5	1.25	DHS 2013–2014; adjusted with ratio	-	Calculated by model
FamPlan					
Contraceptive prevalence rate	3.9	0.5	DHS 2013–2014; adjusted with ratio	-	Calculated by model
LiST					
Baseline child mortality					
Neonatal mortality rate	35		DHS 2013–2014	-	Calculated by model
Infant mortality rate	72		DHS 2013–2014	-	Calculated by model
Under-five mortality rate	121		DHS 2013–2014	-	Calculated by model
Pregnancy					
Antenatal care	42.1	-	DHS 2013–2014; rural estimate, Katanga Regional estimate not available	41.9	Extrapolated/projected DHS
Tetanus toxoid vaccine	37.5	0.87007	DHS 2013–2014; adjusted with ratio	39.9	Extrapolated/projected DHS
Intermittent preventive treatment of malaria during pregnancy	9.2	-	DHS 2013–2014	4.5	Extrapolated/projected DHS
Multiple micronutrition supplementation (iron folate 90+)	3.1	-	DHS 2013–2014	9.9	Extrapolated/projected DHS
Childbirth					
Skilled birth attendance	64.1	-	DHS 2013–2014	62.4	Set to same as institutional delivery; must be greater or equal to institutional delivery
Institutional delivery	63.9	-	DHS 2013–2014	62.4	
Preventive					
Chlorohexadine for postnatal	na	-	LiST default	-	Flatlined
Postnatal care for babies ("clean postnatal practices")	na	-	LiST default	-	Flatlined
Vitamin A supplementation	65.3	0.927557	DHS 2013–2014; adjusted with ratio	68.5	Extrapolated/projected DHS
Improved water source	46.4	0.952772	DHS 2013–2014; adjusted with ratio	57.6	Extrapolated/projected DHS
Water connection in home	0.1	0.111111	DHS 2013–2014; adjusted with ratio	0	Extrapolated/projected DHS
Improved sanitation	11.3	0.61413	DHS 2013–2014; adjusted with ratio	16.9	Extrapolated/projected DHS

RAcE DRC	LiST model baseline inputs—2013			LiST model endline inputs—2016	
	Subnational data	Ratio	Data sources/indicator information	Subnational Data	Data sources/indicator information
Hygienic disposal of child's stools	65.5	-	DHS 2013–2014	-	Flatlined*
Insecticide-treated net ownership (1+ net/hh)	79.9	-	DHS 2013–2014	-	Flatlined†
Vaccines‡					
BCG	83	-	WHO-UNICEF estimate.	80	WHO-UNICEF estimate. Data were entered for each year (2014 = 78%, 2015 = 74%), not interpolated.
DPT vaccine	74	-	WHO-UNICEF estimate.	79	WHO-UNICEF estimate. Data were entered for each year (2014 = 80%, 2015 = 81%), not interpolated.
Hib vaccine	74	-	WHO-UNICEF estimate.	79	WHO-UNICEF estimate. Data were entered for each year (2014 = 80%, 2015 = 81%), not interpolated.
Measles vaccine	76	-	WHO-UNICEF estimate.	77	WHO-UNICEF estimate. Data were entered for each year (2014 = 77%, 2015 = 79%), not interpolated.
Polio vaccine	74	-	WHO-UNICEF estimate.	74	WHO-UNICEF estimate. Data were entered for each year (2014 = 79%, 2015 = 78%), not interpolated.
Pneumococcal vaccine	31	-	WHO-UNICEF estimate.	77	WHO-UNICEF estimate. Data were entered for each year (2014 = 61%, 2015 = 73%), not interpolated.
Rotavirus vaccine	na	-	WHO-UNICEF estimate.	na	WHO-UNICEF estimate.
Curative					
ORS for diarrhea	48.9	1.234848	RAcE 2013 household survey; adjusted with ratio	71.7	RAcE 2016 household survey
Antibiotics for diarrhea	27.5	-	DHS 2013–2014	27.1	Extrapolated/projected DHS
Zinc for diarrhea	2.9	1.208333	RAcE 2013 household survey; adjusted with ratio	58.8	RAcE 2016 household survey
Antibiotics for cough with fast or difficult breathing	17.9	0.454315	RAcE 2013 household survey; adjusted with ratio	53.0	RAcE 2016 household survey
ACT for fever same or next day	5.2	0.305882	RAcE 2013 household survey; adjusted with ratio	49.6	RAcE 2016 household survey

* The extrapolated/projected DHS value was over 100 percent, which was determined unrealistic by ICF and WHO.
† The extrapolated/projected DHS value was over 100 percent, which was determined unrealistic by ICF and WHO. There were no ITN distribution campaigns in the project area from 2013-2016.
‡ WHO-UNICEF estimates of immunization coverage, which were entered for each available year from 2013 to 2016.

Table S2. Intervention coverage indicators and values used in RAcE Malawi LiST analysis

RAcE Malawi	Baseline data inputs—2010 and 2013			Endline inputs—2016	
	Subnational data	Ratio	Data sources/indicator information	Subnational data	Data sources/indicator information
DemProj					
Population (RAcE project area population 2013)	1,906,136	11.18%	Adjusted with ratio	-	Calculated by model
Total fertility rate	6.1	1.18	Adjusted with ratio	-	Calculated by model
AIM					
HIV incidence or prevalence	8.9	0.839623	Adjusted with ratio	-	Calculated by model

RAcE Malawi	Baseline data inputs—2010 and 2013			Endline inputs—2016	
	Subnational data	Ratio	Data sources/indicator information	Subnational data	Data sources/indicator information
FamPlan					
Contraceptive prevalence rate (modern and traditional)	N/A	N/A	DHS 2010	-	Calculated by model
LiST					
Baseline child mortality					
Neonatal mortality rate	34	-	DHS 2010	See results	Calculated by model
Infant mortality rate	73	-	DHS 2010	See results	Calculated by model
Under-five mortality rate	130	-	DHS 2010	See results	Calculated by model
Pregnancy					
Antenatal care	44.9	-	DHS 2010	49.2	DHS 2015-16
Tetanus toxoid vaccine	68.1	0.988389	Adjusted with ratio	71.7	DHS 2015-16
Intermittent preventive treatment of malaria during pregnancy	53.6	0.996283	DHS 2010	63.6	DHS 2015-16
Iron supplementation (90+ days)	30.9	-	DHS 2010	32.2	DHS 2015-16
Childbirth					
Skilled birth attendance	71	-	Set to same as institutional delivery	88.9	DHS 2015-16
Institutional delivery	71	-	DHS 2010	88.9	DHS 2015-16
Breastfeeding					
Early Initiation of breastfeeding (w/in one hour)	94.4	-	DHS 2010	78.3	DHS 2015-16
Preventive					
Postnatal care for babies	N/A	N/A	N/A	N/A	DHS 2015-16
Complementary feeding (both education only and supplementary feeding)	N/A	N/A	N/A	N/A	N/A
Vitamin A supplementation	85.6	0.987313	Adjusted with ratio	64.9	DHS 2015-16
Zinc supplementation	N/A	N/A	N/A	N/A	N/A
Improved water source	77.1	0.967378	Adjusted with ratio	85.2	DHS 2015-16
Water connection in home	1.8	0.272727	Adjusted with ratio	2.3	DHS 2015-16
Improved sanitation	6	0.731707	Adjusted with ratio	53.0	DHS 2015-16
Hand washing	N/A	N/A	N/A	N/A	Flatlined
Hygienic disposal of child's stools	N/A	N/A	N/A	84.4	DHS 2015-16
Insecticide-treated net ownership (1+ net per household)	55.4	-	DHS 2010	55.8	DHS 2015-16
Vaccines					
BCG—1 dose	97.1	0.998971	Adjusted with ratio	97.5	DHS 2015-16
Polio—3 doses	86.7	1.01285	Adjusted with ratio	82.2	DHS 2015-16
DPT vaccine—3 doses	92.8	0.997849	Adjusted with ratio	93.4	DHS 2015-16
Hib vaccine—3 doses	92.8	0.997849	Adjusted with ratio	93.4	DHS 2015-16
HepB—3 doses	92.8	0.997849	Adjusted with ratio	93.4	DHS 2015-16
Measles vaccine	92.5	0.994624	DHS 2010	91.7	DHS 2015-16
Curative					
Maternal sepsis case management	N/A	N/A	N/A	N/A	N/A
Neonatal (all interventions listed)	N/A	N/A	N/A	N/A	N/A
ORS for diarrhea	72.3	1.047826	RAcE Household Survey 2013; Adjusted with ratio	72.6	Flatlined from 2010 to 2013; RAcE Household Survey 2016
Antibiotics for treatment of dysentery	N/A	N/A	N/A	N/A	Flatlined
Zinc for diarrhea	23.8	119.00	RAcE Household Survey 2013; Adjusted with ratio	28.3	Flatlined from 2010 to 2013; RAcE Household Survey 2016

RAcE Malawi	Baseline data inputs—2010 and 2013			Endline inputs—2016	
	Subnational data	Ratio	Data sources/indicator information	Subnational data	Data sources/indicator information
Oral antibiotics for pneumonia	56.5	0.803698	RAcE Household Survey 2013; Adjusted with ratio	66.8	Flatlined from 2010 to 2013; RAcE Household Survey 2016
ACTs for fever within 48 hours	43.5	2.485356	RAcE Household Survey 2013; Adjusted with ratio	N/A	Flatlined 2013 RAcE Household Survey through 2016

N/A = Data not available

Table S3. Intervention coverage indicators and values used in RAcE Mozambique LiST analysis

RAcE Mozambique	LiST model baseline inputs—2013			LiST model endline inputs—2016	
	Subnational data	Ratio	Data sources/indicator information	Subnational data	Data sources/indicator information
DemProj					
Population	4,196,074	20.34%	Project area population estimated by Save the Children; adjusted with ratio	-	Calculated by model
Total fertility rate	6.2	1.05084746	DHS 2011; adjusted with ratio	-	Calculated by model
AIM					
HIV incidence or prevalence	N/A	-	LiST default	-	Calculated by model
FamPlan					
Contraceptive prevalence rate (modern and traditional)	6.9	0.59482759	DHS 2011; adjusted with ratio	-	Calculated by model
LiST					
Baseline child mortality					
Neonatal mortality rate	23	-	DHS 2011	-	Calculated by model
Infant mortality rate	60	-	DHS 2011	-	Calculated by model
Under-five mortality rate	94	-	DHS 2011	-	Calculated by model
Pregnancy					
Antenatal care	38.1	0.75296443	HMIS 2013; adjusted with ratio	21.6	HMIS 2016
Tetanus toxoid vaccine	68.1	1.21824687	HMIS 2013; adjusted with ratio	38.3	HMIS 2016
Intermittent preventive treatment of malaria during pregnancy	30.2	1.54081633	HMIS 2013; adjusted with ratio	38.5	HMIS 2016
Multiple micronutrition supplementation (iron folate 90+)	45.0	0.55624227	HMIS 2013; adjusted with ratio	30.4	HMIS 2016
Childbirth					
Skilled birth attendance	73.6	-	set to same as institutional delivery; cannot be greater	-	set to same as institutional delivery
Institutional delivery	73.6	1.34306569	HMIS 2013; adjusted with ratio	78.2	HMIS 2016
Preventive					
Chlorohexadine for postnatal	N/A	-	LiST default	N/A	Flatlined
Postnatal care for babies ("clean postnatal practices")	N/A	-	LiST default	N/A	Flatlined
Vitamin A supplementation	71.2	0.9557047	DHS 2011; adjusted with ratio	N/A	Flatlined

RAcE Mozambique	LiST model baseline inputs—2013			LiST model endline inputs—2016	
	Subnational data	Ratio	Data sources/indicator information	Subnational data	Data sources/indicator information
Improved water source	42.6	0.83529412	DHS 2011; adjusted with ratio	50.1	Extrapolated/projected DHS
Water connection in home	0.82	0.3037037	DHS 2011; adjusted with ratio	N/A	Flatlined
Improved sanitation	15.1	0.69585253	DHS 2011; adjusted with ratio	N/A	Flatlined
Hygienic disposal of child's stools	78.7	-	DHS 2011	N/A	Flatlined*
Insecticide-treated net ownership (1+ net/hh)	53.7	-	DHS 2011	N/A	Flatlined
Vaccines†					
BCG	93	-	WHO-UNICEF estimate.	95	WHO-UNICEF estimate. Data were entered for each year (2014 = 94%, 2015 = 95%), not interpolated.
DPT3 vaccine	78	-	WHO-UNICEF estimate; LiST default is '0' so could not adjust with ratio	80	WHO-UNICEF estimate. Data were entered for each year (2014 = 79%, 2015 = 80%), not interpolated.
Hib vaccine (Hib3)	78	-	WHO-UNICEF estimate.	80	WHO-UNICEF estimate. Data were entered for each year (2014 = 79%, 2015 = 80%), not interpolated.
Measles vaccine (MCV1)	85	-	WHO-UNICEF estimate.	91	WHO-UNICEF estimate. Data were entered for each year (2014 = 85%, 2015 = 85%), not interpolated.
Polio vaccine	78	-	WHO-UNICEF estimate.	80	WHO-UNICEF estimate. Data were entered for each year (2014 = 79%, 2015 = 80%), not interpolated.
Pneumococcal vaccine (PcV3)	45	-	WHO-UNICEF estimate (no national data for ratio adjustment).	80	WHO-UNICEF estimate. Data were entered for each year (2014 = 73%, 2015 = 80%), not interpolated.
Rotavirus vaccine	N/A	-	WHO-UNICEF estimate.	76	WHO-UNICEF estimate. Data were entered for each year (2014 = N/A, 2015 = 17%), not interpolated.
Curative					
ORS for diarrhea	69.8	1.26909091	RAcE 2013 baseline household survey; adjusted with ratio	69.9	RAcE 2016 endline household survey
Antibiotics for diarrhea	27.3	-	DHS 2011	N/A	Flatlined
Zinc for diarrhea	9.9	-	RAcE 2013 baseline household survey (no national data and LiST default is '0' so could not adjust with ratio)	35.0	RAcE 2016 endline household survey
Antibiotics for cough with fast or difficult breathing	69.9	1.39243028	RAcE 2013 baseline household survey; adjusted with ratio	58.8	RAcE 2016 endline household survey
ACT for fever same or next day	55.0	3.57142857	RAcE 2013 baseline household survey; adjusted with ratio	39.7	RAcE 2016 endline household survey

N/A = Data not available

* The extrapolated/projected DHS value was over 100 percent, which was determined unrealistic by ICF and WHO.
† WHO-UNICEF estimates of immunization coverage, which were entered for each available year from 2013 to 2016.

Table S4. Intervention coverage indicators and values used in RAcE Niger LiST analysis

RAcE Niger	LiST model baseline inputs—2013			LiST model endline inputs—2016	
	Subnational data	Ratio	Data sources/indicator information	Subnational data	Data sources/indicator information
Demproj					
Population	994,904	5.808%	Subnational population from World Vision 2013 grant agreement letter calculated from Niger MSP data; adjusted with ratio	-	calculated by model
Total fertility rate	7.7	1.0131579	Adjusted with ratio; DHS	-	calculated by model
AIM					
HIV incidence or prevalence	N/A		LiST default	-	calculated by model
Famplan					
Contraceptive prevalence rate (modern and traditional)	9.3	0.6690647	Adjusted with ratio; DHS	-	calculated by model
LiST					
Baseline child mortality					
Neonatal mortality rate	28		DHS 2012	-	results calculated by model
Infant mortality rate	56		DHS 2012	-	results calculated by model
Under-five mortality rate	137		DHS 2012	-	results calculated by model
Pregnancy					
Antenatal care	14.7	0.45	HMIS 2013; adjusted with ratio	17.1	HMIS 2016
Tetanus toxoid vaccine	28.2	0.57	HMIS 2013; adjusted with ratio	38.4	HMIS 2016
Intermittent preventive treatment of malaria during pregnancy	22.2	0.64	HMIS 2013; adjusted with ratio	38.3	HMIS 2016
Multiple micronutrition supplementation (iron folate 90+)	22.5	0.28	HMIS 2013; adjusted with ratio	42.5	HMIS 2016
Childbirth					
Skilled birth attendance	N/A	-	set to same as institutional delivery	56.8	set same as institutional delivery
Institutional delivery	44.4	1.49	HMIS 2013; adjusted with ratio	56.8	HMIS 2016
Preventive					
Chlorohexadine for postnatal	18.8	-	no DHS data available; used HMIS 2013	18.3	HMIS 2016
Postnatal care for babies ("clean postnatal practices")	16.8	-	DHS 2012	N/A	flatlined
Vitamin A supplementation	53.5	0.899	DHS 2012; adjusted with ratio	40.4	extrapolated/projected DHS
Improved water source	48.7	0.727	DHS 2012; adjusted with ratio	62.9	extrapolated/projected DHS
Water connection in home	0.14	0.052	DHS 2012; adjusted with ratio	0.2	extrapolated/projected DHS
Improved sanitation	3.1	0.333	DHS 2012; adjusted with ratio	4.6	extrapolated/projected DHS
Hygienic disposal of child's stools	10.4	-	DHS 2012	14.8	extrapolated/projected DHS
Insecticide-treated net ownership (1+ net/hh)	63.5	-	DHS 2012	76.6	extrapolated/projected DHS
Vaccines					

RAcE Niger	LiST model baseline inputs—2013			LiST model endline inputs—2016	
	Subnational data	Ratio	Data sources/indicator information	Subnational data	Data sources/indicator information
BCG	37	-	WHO-UNICEF estimate.	77	WHO-UNICEF estimate. Data were entered for each year (2014 = 76%, 2015 = 77%), not interpolated.
DPT vaccine (DPT3)	67	-	WHO-UNICEF estimate.	67	WHO-UNICEF estimate. Data were entered for each year (2014 = 68%, 2015 = 65%), not interpolated.
Hib vaccine (Hib3)	67	-	WHO-UNICEF estimate.	67	WHO-UNICEF estimate. Data were entered for each year (2014 = 68%, 2015 = 65%), not interpolated.
Measles vaccine (MCV1)	76	-	WHO-UNICEF estimate.	74	WHO-UNICEF estimate. Data were entered for each year (2014 = 72%, 2015 = 73%), not interpolated.
Polio vaccine (Pol3)	56	-	WHO-UNICEF estimate.	67	WHO-UNICEF estimate. Data were entered for each year (2014 = 67%, 2015 = 65%), not interpolated.
Pneumococcal vaccine (PcV3)	n/a	-	WHO-UNICEF estimate.	64	WHO-UNICEF estimate. Data were entered for each year (2014 = 13%, 2015 = 49%), not interpolated.
Rotavirus vaccine (RotaC)	n/a	-	WHO-UNICEF estimate.	61	WHO-UNICEF estimate. Data were entered for each year (2014 = 19%, 2015 = 47%), not interpolated.
Curative					
ORS for diarrhea	61.7	1.393	2013 RAcE household survey; adjusted with ratio	78.4	2016 RAcE household survey
Antibiotics for diarrhea	6.1	0.504	used ratio HMIS	4.4	HMIS 2016
Zinc for diarrhea	30.4	N/A	2013 RAcE household survey; default LiST data at '0' so no ratio adjustment	72.6	2016 RAcE household survey
Antibiotics for cough with fast or difficult breathing	44.6	0.840	2013 RAcE household survey; adjusted with ratio	46.2	2016 RAcE household survey
ACT for fever same or next day	41.9	3.612	2013 RAcE household survey; adjusted with ratio	58.4	2016 RAcE household survey

N/A= Data not available

Table S5. Intervention coverage indicators and values used in RAcE Abia State LiST Analysis

RAcE Abia State, Nigeria	Baseline data inputs—2013			Endline data inputs—2016	
	Subnational data	Ratio	Data sources/indicator information	Subnational data	Data sources/indicator information
DemProj					
Population	1,268,738	0.90%	Project area population (in CORP catchment areas) in 2015 provided by SFH; adjusted with ratio	-	Calculated by model
Total fertility rate	4.2	0.763636	DHS 2013; Abia State; adjusted with ratio	-	Calculated by model
AIM					
HIV incidence or prevalence	N/A	-	LiST default	-	Calculated by model
FamPlan					
Contraceptive prevalence rate (modern and traditional)	33.4	2.0875	DHS 2013; Abia State; Adjusted with ratio	-	Calculated by model
LiST					
Baseline child mortality					
Neonatal mortality rate	37	-	DHS 2013; South East	-	Calculated by model
Infant mortality rate	82	-	DHS 2013; South East	-	Calculated by model
Under-five mortality rate	131	-	DHS 2013; South East	-	Calculated by model
Pregnancy					
Antenatal care	38.2	-	DHS 2013, national-rural estimate lowest level of disaggregation available	40.5	Extrapolated/ projected DHS
Tetanus toxoid vaccine	88.7	1.832645	Adjusted with ratio	95.3	Extrapolated/ projected DHS
Intermittent preventive treatment of malaria during pregnancy	21.7	-	DHS 2013	31.5	Extrapolated/ projected DHS
Multiple micronutrition supplementation (iron folate 90+)	84.7	-	DHS 2013	100	Projected value using trend is more than 100%
Childbirth					
Skilled birth attendance	77.2	-	DHS 2013	74.4	Extrapolated/ projected DHS
Institutional delivery	72.8	-	DHS 2013	72.1	Extrapolated/ projected DHS
Preventive					
Chlorohexidine for postnatal	N/A	-	LiST default	0	Flatlined
Postnatal care for babies ("clean postnatal practices")	N/A	-	LiST default	14.0	Flatlined
Vitamin A supplementation	65.9	1.595642	Ratio adjusted to more than 100%, so used 100%	88.3	Extrapolated/ projected DHS
Improved water source	62.6	1.033003	Adjusted with ratio	62.6	Extrapolated/ projected DHS
Water connection in home	na	-	LiST default	3.4	Flatlined
Improved sanitation	42.5	1.41196	Adjusted with ratio	42.5	Extrapolated/ projected DHS
Hygienic disposal of child's stools	64.6	-	DHS 2013	67.1	Extrapolated/ projected DHS
Insecticide-treated net ownership (1+ net/hh)	59.6	-	DHS 2013	51.9	MIS 2015; flatlined in 2016
Vaccines*					

RAcE Abia State, Nigeria	Baseline data inputs—2013			Endline data inputs—2016	
	Subnational data	Ratio	Data sources/indicator information	Subnational data	Data sources/indicator information
BCG	57	-	WHO-UNICEF estimate.	64	WHO-UNICEF estimate. Data were entered for each year (2014 = 64%, 2015 = 64%), not interpolated.
DPT vaccine (DPT3)	46	-	WHO-UNICEF estimate.	49	WHO-UNICEF estimate. Data were entered for each year (2014 = 49%, 2015 = 49%), not interpolated.
Hib vaccine (Hib3)	30	-	WHO-UNICEF estimate.	49	WHO-UNICEF estimate. Data were entered for each year (2014 = 49%, 2015 = 49%), not interpolated.
Measles vaccine (MCV1)	46	-	WHO-UNICEF estimate.	51	WHO-UNICEF estimate. Data were entered for each year (2014 = 51%, 2015 = 51%), not interpolated.
Polio vaccine (Pol3)	46	-	WHO-UNICEF estimate.	49	WHO-UNICEF estimate. Data were entered for each year (2014 = 49%, 2015 = 49%), not interpolated.
Pneumococcal vaccine (PcV3)	N/A	-	WHO-UNICEF estimate.	26	WHO-UNICEF estimate. Data were entered for each year (2014 = na%, 2015 = 13%), not interpolated.
Rotavirus vaccine (RotaC)	N/A	-	WHO-UNICEF estimate.	N/A	WHO-UNICEF estimate, indicated data were not available. Flatlined.
Curative					
ORS for diarrhea	31.4	0.931751	RAcE household survey 2014; adjusted with ratio	55.4	RAcE 2017 household survey
Antibiotics for diarrhea	37.6	-	DHS 2013	46.2	Extrapolated/ projected DHS
Zinc for diarrhea	7.2	3.130435	RAcE household survey 2014; adjusted with ratio	42.0	RAcE 2017 household survey
Antibiotics for cough with fast or difficult breathing	8.6	0.235616	RAcE household survey 2014; adjusted with ratio	35.5	RAcE 2017 household survey
ACT for fever same or next day	15.3	2.55	RAcE household survey 2014; adjusted with ratio	38.3	RAcE 2017 household survey

N/A = Data not available

*WHO-UNICEF estimates of immunization coverage, which were entered for each available year from 2013 to 2016.

Table S6. Intervention coverage indicators and values used in RAcE Niger State LiST analysis

RAcE Niger State, Nigeria	LiST Model Baseline Inputs – 2013			LiST Model Endline Inputs – 2016	
	Subnational data	Ratio	Data sources/indicator information	Subnational data	Data sources/indicator information
DemProj					
Population	814,845	0.58%	RAcE project area population estimated by Malaria Consortium	-	Calculated by model
Total fertility rate	6.1	1.10909	DHS 2013; Niger State; adjusted with ratio	-	Calculated by model
AIM					
HIV incidence or prevalence	N/A	N/A	LiST default	-	Calculated by model
FamPlan					
Contraceptive prevalence rate	6.6	0.4125	DHS 2013; Niger State; adjusted with ratio	-	Calculated by model
LiST					
Baseline child mortality					
Neonatal mortality rate	35	-	DHS 2013; North Central	-	Calculated by model
Infant mortality rate	66	-	DHS 2013; North Central	-	Calculated by model
Under-five mortality rate	100	-	DHS 2013; North Central	-	Calculated by model
Pregnancy					
Antenatal care	38.2		DHS 2013; national rural data was lowest level of disaggregation available in DHS	40.5	Extrapolated/projected DHS
Tetanus toxoid vaccine	51.4	1.06198	Adjusted with ratio	54.8	Extrapolated/projected DHS
Intermittent preventive treatment of malaria during pregnancy	34.5	-	DHS 2013	51.1	Extrapolated/projected DHS
Multiple micronutrition supplementation (iron folate 90+)	76.8	-	DHS 2013	100	Extrapolated/projected DHS
Childbirth					
Skilled birth attendance	28.6	-	DHS 2013	20.1	Extrapolated/projected DHS
Institutional delivery	25.3	-	DHS 2013	15.9	Extrapolated/projected DHS
Preventive					
Chlorohexadine for postnatal	N/A	-	LiST default	N/A	Flatlined
Postnatal care for babies ("clean postnatal practices")	N/A	-	LiST default	N/A	Flatlined
Vitamin A supplementation	33.4	0.80872	Adjusted with ratio	38.0	Extrapolated/projected DHS
Improved water source	48.1	0.79373	Adjusted with ratio	48.1	Flatlined
Water connection in home	N/A		LiST default	N/A	Flatlined
Improved sanitation	16.6	0.5515	Adjusted with ratio	N/A	Flatlined
Hygienic disposal of child's stools	20.9	-	DHS 2013	13.6	Extrapolated/projected DHS
Insecticide-treated net ownership (1+ net/hh)	49.4	-	DHS 2013	61.2	MIS 2015; flatlined in 2016
Vaccines*					
BCG	57	-	WHO-UNICEF estimate.	64	WHO-UNICEF estimate. Data were entered for each

RAcE Niger State, Nigeria	LiST Model Baseline Inputs – 2013			LiST Model Endline Inputs – 2016	
	Subnational data	Ratio	Data sources/indicator information	Subnational data	Data sources/indicator information
					year (2014 = 64%, 2015 = 64%), not interpolated.
DPT vaccine (DPT3)	46	-	WHO-UNICEF estimate.	49	WHO-UNICEF estimate. Data were entered for each year (2014 = 49%, 2015 = 49%), not interpolated.
Hib vaccine (Hib3)	30	-	WHO-UNICEF estimate.	49	WHO-UNICEF estimate. Data were entered for each year (2014 = 49%, 2015 = 49%), not interpolated.
Measles vaccine (MCV1)	46	-	WHO-UNICEF estimate.	51	WHO-UNICEF estimate. Data were entered for each year (2014 = 51%, 2015 = 51%), not interpolated.
Polio vaccine (Pol3)	46	-	WHO-UNICEF estimate.	49	WHO-UNICEF estimate. Data were entered for each year (2014 = 49%, 2015 = 49%), not interpolated.
Pneumococcal vaccine (PcV3)	N/A	-	WHO-UNICEF estimate.	26	WHO-UNICEF estimate. Data were entered for each year (2014 = na%, 2015 = 13%), not interpolated.
Rotavirus vaccine (RotaC)	N/A	-	WHO-UNICEF estimate.	N/A	WHO-UNICEF estimate, indicated data were not available. Flatlined.
Curative					
ORS for diarrhea	68.2	2.02374	RAcE 2013 household survey; Adjusted with ratio	88.3	RAcE 2017 household survey
Antibiotics for diarrhea	40	-	DHS 2013	51.1	Extrapolated/projected DHS
Zinc for diarrhea	15	6.52174	RAcE 2013 household survey; Adjusted with ratio	77.0	RAcE 2017 household survey
Antibiotics for cough with fast or difficult breathing	28.6	0.78356	RAcE 2013 household survey; Adjusted with ratio	60.5	RAcE 2017 household survey
ACT for fever same or next day	27.4	4.56667	RAcE 2013 household survey; Adjusted with ratio	64.5	RAcE 2017 household survey

N/A = Data not available
*WHO-UNICEF estimates of immunization coverage, which were entered for each available year from 2013 to 2016.

Estimated Child Lives Saved per Year and by Treatment Interventions for Each RAcE Project Area

Table S7. Estimated child lives saved per year by treatment interventions in the DRC

RAcE DRC	2013	2014	2015	2016	Total	Percentage intervention treatment by CHWs	Estimated lives saved by CHW-provided treatment
Total lives saved among children 1–59 months (all interventions)	0	316	650	963	1,929		
**Intervention**	**Estimated lives saved**						
ORS	0	71	143	217	431	90%	388
Zinc for treatment of diarrhea	0	24	49	75	148	95%	141
Oral antibiotics for pneumonia	0	83	163	247	493	97%	478
ACTs for treatment of malaria	0	120	245	378	743	97%	721
				Total	***1,815***	**-**	***1,728***

Table S8. Estimated child lives saved per year by iCCM interventions in Malawi

RAcE Malawi	2013*	2014	2015	2016	Total	Percentage intervention treatment by CHWs	Estimated lives saved by CHW-provided treatment
Total lives saved among children 1–59 months (all interventions)	189	292	395	477	1,353		
**Intervention**	**Estimated lives saved**						
ORS for diarrhea	0	3	6	10	19	54%	10
Zinc for diarrhea	0	7	15	23	45	52%	23
Oral antibiotics for pneumonia	0	81	167	260	508	36%	183
ACT for malaria	0	0	0	0	0	49%	0
Total lives saved by year	*0*	*91*	*188*	*293*			
				Total	***572***	**-**	***216***

Table S9. Estimated child lives saved per year by treatment interventions in Mozambique

RAcE Mozambique	2013*	2014	2015	2016	Total
Total lives saved among children 1–59 months (all interventions)	0	-59	-129	-235	-423
**Treatment intervention**	**Estimated lives saved**				
ORS	0	3	6	8	17
Zinc for treatment of diarrhea	0	43	85	123	251
Oral antibiotics for pneumonia	0	-149	-301	-462	-912
ACTs	0	-273	-554	-848	-1,675
				Total	-2,319

Table S10. Estimated child lives saved per year by treatment interventions in Niger

RAcE Niger	2013*	2014	2015	2016	Total	Percentage intervention treatment by CHWs	Estimated lives saved by CHW-provided treatment
Total lives saved among children 1–59 months (all interventions)	0	200	475	793	1,468		
**Intervention**	**Estimated lives saved**						
ORS	0	92	188	288	568	86%	488
Zinc for treatment of diarrhea	0	29	59	91	179	89%	159
Oral antibiotics for pneumonia	0	9	18	27	54	55%	30
ACTs	0	55	109	163	327	88%	288
				**Total**	**1,128**	**-**	**965**

Table S11. Estimated number of child lives saved per year by treatment interventions in Abia State, Nigeria

RAcE Abia State	2013*	2014	2015	2016	Total	Percentage intervention treatment by CHWs	Estimated lives saved by CHW-provided treatment
Total lives saved among children 1–59 months (all interventions)	0	273	523	777	1,573		
**Intervention**	**Estimated lives saved**						
ORS for treatment of diarrhea	0	60	119	178	357	59%	211
Zinc for treatment of diarrhea	0	16	33	49	98	72%	71
Oral antibiotics for treatment of pneumonia	0	81	158	233	472	74%	349
ACT for treatment of malaria	0	77	158	245	480	70%	336
				Total	***1,407***	**-**	***967***

Table S12. Estimated child lives saved per year by iCCM interventions in Niger State, Nigeria

RAcE Niger State, Nigeria	2013*	2014	2015	2016	Total	Percentage intervention treatment by CHWs	Estimated lives saved by CHW-provided treatment
Total lives saved among children 1–59 months (all interventions)	0	235	474	714	1,423		
**Intervention**	**Estimated lives saved**						
ORS for treatment of diarrhea	0	46	91	136	273	85%	232
Zinc for treatment of diarrhea	0	15	29	44	88	89%	78
Oral antibiotics for treatment of pneumonia	0	68	136	203	407	78%	317
ACT for treatment of malaria	0	86	169	251	506	86%	435
				Total	***1,274***	**-**	***1,062***
